# Ovarian cancer stem cells: Critical roles in anti-tumor immunity

**DOI:** 10.3389/fgene.2022.998220

**Published:** 2022-11-10

**Authors:** Jing Ding, Yan Zhang, Yan Che

**Affiliations:** NHC Key Lab of Reproduction Regulation (Shanghai Institute for Biomedical and Pharmaceutical Technologies), Fudan University, Shanghai, China

**Keywords:** cancer stem cells, ovarian cancer, immune escape, cancer-microenvironment, molecular targeted therapy

## Abstract

Ovarian cancer is a significant cause of cancer-related mortality in women. Over the past 3 decades, there has been a high incidence of recurrent chemoresistant disease, despite the relative effectiveness of current treatment strategies. This is partly attributed to cancer stem cells (CSC), a subpopulation that has acquired stem cell properties that allow these cells to evade standard chemotherapy and cause disease recurrence. Therefore, there is an urgent need for basic knowledge about CSC to develop innovative therapeutic approaches for ovarian cancer. These CSC subpopulations have been identified in ovarian cancer cell lines, tumors or ascites, and findings suggest that ovarian CSCs may be as heterogeneous as the disease itself. CSCs regulate the phenotype and function of immune cells involved in antitumor immunity, so a better understanding of the signaling pathways that interact between CSCs, immune cells and tumor cells will pave the way for the clinical application of CS in cancer immunotherapy. This review will focus on the markers currently used to identify and isolate these cells summarize current knowledge on the molecular and cellular mechanisms responsible for CSC-dependent regulation of antitumor immune responses. We will discuss the signaling pathways involved in CSC survival, replication, and differentiation as well as potential therapeutic targeting strategies.

## Introduction

Ovarian cancer (OC) is the third most common gynecologic malignancy worldwide and the leading cause of gynecologic oncology-related deaths worldwide (Kocarnik et al., 2022). Globally, more than 300,000 new cases of ovarian cancer are diagnosed each year and 18,000 patients die from their disease (Ferlay et al., 2019). Due to the lack of typical clinical signs, 67% of patients are already in stages III and IV at the time of first diagnosis (Yang et al., 2020). Currently, initial tumor reduction surgery combined with platinum-based chemotherapy is the standard of care for advanced OC. However, 80% of patients with advanced OC eventually relapse and become resistant to platinum-based therapy; therefore, there is an urgent need for innovation and development of effective therapies (Kuroki and Guntupalli, 2020).

Ovarian cancer is an immunogenic tumor and multiple antigens have been identified in recent years. Some of the strongest evidence linking anti-tumor immunity to cancer has been demonstrated in ovarian cancer (Morand et al., 2021). Understanding how the immune response is activated in ovarian cancer is a prerequisite for the development of immunotherapies. Key to the interaction between tumor and immune cells is the production, expression and release of tumor-associated antigen (TAA) (Alfei et al., 2021). TAA is subsequently phagocytosed and processed by dendritic cells (DCs) and, after delivery to initial CD4 T^+^, CD8 T^+^ cells *via* major histocompatibility complex (MHC) molecules, can elicit a host-specific T-cell immune response in the organism (Alfei et al., 2021). It was found that CD8 T^+^ cells can recognize HER2/neu positive tumor cells in ovarian cancer ascites (Ioannides et al., 1993). In addition, there are other TAAs in ovarian cancer, such as CA125 (Chauhan et al., 2006), folate receptor (FR)-α (Peoples et al., 1998), CA153 (Chauhan et al., 2006), and human epithelial protein HE-4 (Drapkin et al., 2005). In the tumor microenvironment, macrophages occupy 30–50% of the infiltrating immune cells (Curiel et al., 2003). The tumor microenvironment strongly polarizes macrophage differentiation while generating tumor-associated macrophages (TAM), and B7-H4+ macrophages can suppress specific T cell immune responses (Kryczek et al., 2006). Natural killer (NK) cell-derived perforin forms pores in the membranes of tumor cells, allowing granzyme B to enter the cytoplasm and induce apoptosis by cleaving key intracellular substrates that control the survival of cancer cells (Kaschek et al., 2021). CA125 binds to the killer immunoglobulin-like receptors (KIR) siglec-9, thereby protecting itself from NK-mediated cell lysis (Belisle et al., 2010). NK cells express programmed death ligand (PDL), which activates the mitochondrial apoptotic pathway in tumor cells by binding to programmed death receptor (PD) (Hamilton and Plangger, 2021). Ovarian cancer cells express programmed death ligand 1 (PDL-1) that binds to programmed death (PD-1) of CD8^+^ T cells and subsequently impairs the effector function of lymphocytes (Matsuzaki et al., 2010). Immune checkpoint inhibitor like PD-1 blockade was only approved for those patients with mistmach repair deficiency (MMRd) or high microsatellite instability (MSI-H) tumors and most OC does not respond to immune checkpoint inhibitors (André et al., 2020). In addition to effector T cells, macrophages, and NK cells, immunosuppressive CD4^+^FoxP3^+^ Treg cells (Tregs), and myeloid-derived suppressor cells (MDSC) also promote tumor growth and progression (Curiel et al., 2004). The association of Treg cells with a high risk ratio of death and reduced survival time has been well documented in ovarian cancer (Curiel et al., 2004). Treg cells mediate immunosuppression mainly through intercellular contacts with DC cells or effector cells or by secreting immunosuppressive cytokines, including IL-10, IL-35 and TGF-β. Treg contribute to DC tolerance, which further reduces the activation and proliferation of effector T cells (Oleinika et al., 2013). MDSC are immature bone marrow cells with immunosuppressive properties and have been found in both ovarian cancer patients and ovarian cancer mouse models (Bak et al., 2008). MDSC induce arginase 1 (ARG-1) and inducible nitric oxide synthase (iNOS) activity leads to downregulation of the CD3-zeta chain, which inhibits effector T cell activation (Ezernitchi et al., 2006). Increased NO levels block IL-2 receptor signaling and alter Ag recognition by nitrating the TCR (Nagaraj et al., 2007). Different immune cells affect the oncogenesis of tumor immunity in opposite directions, therefore modulation of their phenotype and function represents a potential strategy to enhance ovarian cancer treatment.

Ovarian cancer stem cells (OCSC) are self-renewable pluripotent stem cells that have an important role in cancer development, progression, metastasis and recurrence, as well as in resistance to radiation and chemotherapy (Konrad et al., 2017). Current evidence suggests that tumor stem cells may develop a mechanism to evade immune attack, and it remains unknown whether OCSC have the same immune escape mechanism, and these cells are a major driver of tumor formation, progression, metastasis and apoptotic resistance to chemotherapy and radiotherapy (Keyvani et al., 2019). Ovarian cancer is usually associated with peritoneal ascites, in which spheroids are present in tumor cells that survive and proliferate even in a non-adherent state (Mihanfar et al., 2019). A recent study showed that chemotherapy usually leaves behind an OCSC-like cellular entity and that these cells are more aggressive and induce disease recurrence (Rizzo et al., 2011). Similarly, recurrent ovarian cancer is rich in OCSCs, suggesting that OCSCs may contribute to cancer recurrence (Steg et al., 2012). Residual OCSCs surviving chemotherapy may provide a favorable microenvironment to promote the growth of residual cells. Resistance to loss-of-nest apoptosis is a key feature of these stem cells, and in normal tissues after injury, tissue-specific stem cells expand to initiate repair prior to their differentiation. Once the tissue is repaired, the stem cells return to a quiescent state (Al-Alem et al., 2019). However, in tumor tissues, tissue injury (caused by surgery or chemotherapy) causes OCSCs to interact with the local tumor microenvironment and release various inflammatory cytokines, chemokines, and matrix metalloproteinases that induce invasion and spread to distant organs in the body, and can alter the phenotype and function of immune cells (Ahmed et al., 2018). Therefore, in this review, we describe the role of OCSCs in ovarian cancer immunity and discuss its molecular and cellular mechanisms in the problems of recurrence and chemoresistance.

## Identification of ovarian cancer stem cells

Ovarian cancer stem cells represent a specific group of cells that are cellularly and molecularly heterogeneous, capable of self-renewal and reflecting pluripotency. Bapat et al. reported for the first time the isolation and characterization of ovarian cancer stem cells, where a tumorigenic clone was isolated from a mixed cell population in the ascites of ovarian cancer patients, exhibiting anchorage-independent growth and forming spheroids. These cells express the cytokine CD117 and are also capable of continuously generating new tumors if transplanted into the peritoneal cavity of mice (Bapat et al., 2005). Zhang et al. isolated and characterized ovarian cancer-initiating cells from primary tumors of five patients that were fully capable of reconstituting their original tumor hierarchy *in vivo*. The main markers identified were CD117 and CD44. These spheroid-forming cells are resistant to resistant to conventional chemotherapy and can form xenograft tumors with the same phenotype (Zhang et al., 2008). In another study, Chang et al. identified and isolated cells with CSC characteristics based on the SKOV3 human ovarian cancer cell line and found that CD24^-/low^ SKOV3 cells exhibited stem cell-like characteristics such as high clonogenic capacity, enriched SP cell ratio, and tumorigenesis (Chang et al., 2020). A major limitation of this study is that it was limited to the study of cancer cell lines, which carry many variants due to culture conditions.

The above study demonstrated the presence of multiple cell populations in ovarian tumors, and researchers have identified various markers and combinations of markers suggestive of ovarian cancer stem cells. For example, CD24, CD44^+^/CD24^-^, CD117/c-kit, CD44^+^/CD117^+^, *etc.* have been proposed (Table 1). However, the reported markers are highly variable, which may be related to the different stages of the CSC hierarchy or potential differences in tumor origin.

**TABLE 1:** Cancer stem cell markers used to isolate ovarian cancer stem cells.

Surface marker	Description	Experimental design	The distinctive feature of these cells	References
CD24	Transmembrane glycoprotein	Caov3	Metastasis and chemoresistance through the induction of epithelial to mesenchymal transition *via* Akt-ERK signaling mechanism	Nakamura et al. (2017)
CD44^+^/CD24^−^	CD44: Hyaluronate receptor	SKOV3 and OV90, Cancer cell isolated from ascites of ovarian cancer patients	Predictor of chemoresistance, relapse, and poor prognosis	Meng et al. (2012)
CD117/c-kit	Receptor/Oncoprotein having tyrosine kinase activity	Paraffn-embedded specimens of human serous ovarian carcinoma	Indicator of chemoresistance	Raspollini et al. (2004)
CD133	Transmembrane glycoprotein	Cancer cell lines and cells isolated from ascitic fuid of ovarian cancer patients	Indicator of tumorigenicity and its expression is modulated by epigenetics	Baba et al. (2009)
ALDH1A1	Intracellular enzyme, one of 17 isoforms of ALDH	Several cancer cell lines and primary xenograft developed from omental tissue of metastatic ovarian cancer patients	Predictor of tumor initiation, identifcation of chemoresistant cells	Landen et al. (2010)
CD44^+^/CD117^+^	CD117: Stem cell factor receptor	Xenograft experiment	Indicator of greater tumorigenicity	Zhang et al. (2008)
SP cells	Having dye exclusion property	H2B-GFP transgenic mice models	Identifcation and characterization of ovarian cancer stem cells	Hu et al. (2010)
CD44^+^/MyD88^+^	MYD88: Innate immune signal transduction	Ascites sample from advanced ovarian cancer patients	Maintenance of cell survival and chemoresistance *via* TLR4-MyD88 and NF kappa B pathway	Kusumbe and Bapat, (2009)
	adaptor			
CD34^+^	Transmembrane phosphoglycoprotein	Xenograft tumor	Role in angiogenesis	Alvero et al. (2009)
CD105, CD44	CD105: Type I membrane glycoprotein; CD106:vascular cell adhesion molecule-1	OVCAR3	progression of disease, relapse, and chemoresistance	Zhang et al. (2019)
CD106				
CD34^+^	Transmembrane phosphoglycoprotein	Xenograft tumor	Role in angiogenesis	Alvero et al. (2009)
CD44^−^EpCAM	EpCAM: Type I transmembrane glycoprotein	OVCAR8, SKOV3, OCC1	Cell growth and apoptosis	Zheng et al. (2017)
		ES2, and HEK293		

## OCSC-dependent immune escape

Burnet introduced the concept of tumor immunosurveillance in 1970, whereby the immune system recognizes cancer cells and/or precancerous cells and eliminates them before they can cause harm. The immune system can effectively protect the host from microbial pathogens (Burnet, 1970). Despite tumor immune surveillance, tumors do develop in the presence of a functioning immune system, so this concept was subsequently superseded by the tumor immune editing hypothesis. The tumor immune editing hypothesis is a more complete explanation of the role of the immune system in tumor development in which three phases, elimination, homeostasis, and escape shape tumor immunity (Dunn et al., 2002).

Understanding how OCSCs evade the immune system is relevant for OCSCs to evade standard chemotherapy and lead to disease recurrence. The CD24 expressed on the surface of OCSCs binds sialic-acid-binding Ig-like lectin 10 (Siglec-10) and thus protects itself from macrophage action (Barkal et al., 2019). In addition, ovarian cancer stem cells have constitutive NF-κB activity and sustained activation of NF-κB signaling allows tumor cells to avoid apoptosis while inducing a chronic inflammatory response in the tumor microenvironment (Jo et al., 2020). Yin et al. found that ovarian cancer stem cells (type I/CD44^+^) create a pro-inflammatory and anti-apoptotic environment by activating the NF-κB signaling pathway through low expression of Twist1 environment and thus transformed to ovarian cancer cells. Among the mechanisms by which Twist1 promotes these changes is through the expression of mi199a/214 located in the Dnm3 gene (Yin et al., 2010).

Immune cells also play an important role in OCSCs-dependent immune escape. During ovarian cancer progression, different types of T cells are recruited to the tumor lesion for tumor immune response, including CD4^+^ cells, CD8^+^ T cells (Bamias et al., 2008). It has been demonstrated that OCT4^+^MYC^+^ NANOG^+^ cells constitute OCSCs and that most patients with ovarian tumors have naturally occurring memory T cells specific for OCT4 in the peripheral blood (Looijenga et al., 2003). However, Di et al. detected low expression of CD4^+^ and CD8^+^ T cells specific for OCT4 in the blood and ascites of ovarian cancer patients, which may reflect another mechanism by which ovarian tumors evade immune surveillance (Di et al., 2013). In addition, loss of MHC molecules is frequently observed in cancer cells, making tumor cells resistant to T cell-mediated cytotoxicity (Lu et al., 2011). However, γδ T cells exhibit effective MHC-independent lytic activity against different tumor cells, suggesting an important role for γδ T cells in defense. Lai et al. found that γδ T cells have cytotoxic effects on OCSCs and can effectively kill OCSCs by producing IL-17. γδ T cells also induce HLA-DR, B7-1 and B7-2 expression, which may promote antigen expression in tumor cells and contribute to tumor cell recognition by the immune system (Lu et al., 2011). Deng et al. investigated the effect of OCSCs on macrophage differentiation, and they demonstrated that OCSCs promote anti-inflammatory/pro-tumor M2 macrophage polarization through NF-κB and PPARγ pathways (Deng et al., 2015). Finally, MDSCs are myeloid cells expressing GR1 and CD11b that can induce T cell apoptosis by depleting T lymphocytes of nutrients required for survival, such as arginine and cysteine (Kumar et al., 2016). Cui et al. found that MDSCs inhibit T cell activation and enhance CSC gene expression, identifying the MDSCs-microRNA101-CtBP2-immune-related cellular network of stem cell core genes (Cui et al., 2013).

## The messenger role of OCSC in tumor metastasis

During ovarian cancer progression, the tumor microenvironment, including stromal cells, endothelial cells, infiltrating immune cells and extracellular matrix, OCSCs and the tumor microenvironment interact, resulting in the presence of many immunosuppressive or soluble cytokines in the tumor microenvironment (Luo et al., 2016). The microenvironment not only maintains OCSC at the stem cell state, but also directly influences the differentiation of normal cells to OCSC and informs epithelial mesenchymal transition (EMT), which leads to high potential for invasion and metastasis formation in cancer cells (Li and Neaves, 2006). In the series of tumor invasion and metastasis, the most complicated procedure arises when the metastatic tumor cells reach the parenchymal tissue, then proliferate and form clones in the new environment, and finally shape the tumor mass (Mani et al., 2008). Due to the tumor initiation ability of OCSC, active OCSC tend to form multiple micrometastases *in vivo*. A more thorough study of the mechanisms involved in the metastatic process of OCSC is essential to lead to new therapeutic strategies aimed at the eradication of OCSC.

**FIGURE 1:**
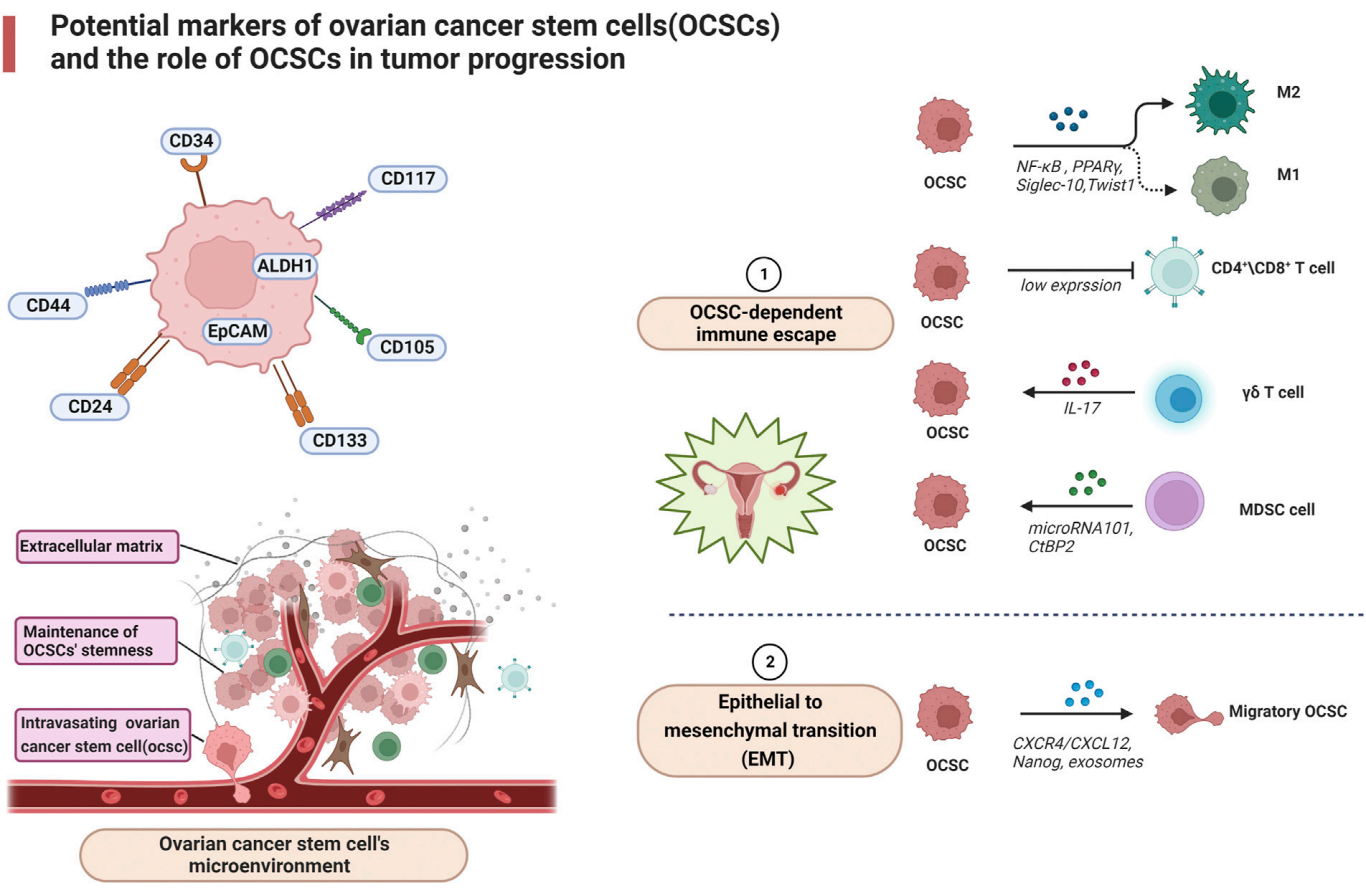
Potential markers of ovarian cancer stem cells (OCSCs) and the role of OCSCs in tumor progression.

The chemokine/receptor complex: C-X-C motif chemokine receptor 4/(CXCR4) from the extracellular matrix is highly expressed in ovarian cancer cells and is associated with ovarian cancer metastasis (Balkwill, 2004). The chemokine/receptor complex: C-X-C motif chemokine receptor four/CXCR4 blockers stimulate antitumor immunity by decreasing T regulatory cell infiltration and increasing T helper cell and cytotoxic T cell infiltration into the tumor microenvironment of mice bearing tumors and ascites fluid (Gil et al., 2014). In addition, CXCL12 has been shown to recruit suppressive MDSCs and pDCs at tumor sites (Obermajer et al., 2011) and induce intra-tumor regulatory T cell (Tregs) localization (Curiel et al., 2004), thereby impeding the immune machinery of ovarian cancer destruction. Extracellular vesicles released by ovarian cancer stem cells signal through lipids, proteins, DNA, mRNA and micro RNA with other components of the tumor microenvironment, such as stromal cells and extracellular matrix. For immune cells, exosomes of malignant ascites origin may induce apoptosis of DCs (peripheral blood lymphocytes) and PBMCs (dendritic cells) precursors (Peng et al., 2011) and may contain immunosuppressive factors such as TGF-β1 and IL-10, suggesting that exosomes may be involved in supporting immune evasion in OvCa (Szajnik et al., 2010). Exosomes may also act as carriers to transfer miR222-3p into macrophages, inducing M2-like polarization of macrophages to produce TAM (Ying et al., 2016). Furthermore, exosomes containing miR-21, miR-103, miR-205 and miR-200 have been associated with poor outcomes in patients with ovarian cancer. Due to its great diagnostic and therapeutic potential, this extracellular vesicle is considered one of the most recently investigated targets for the treatment of tumors (Cheng et al., 2017). Nanog, as a stemness marker, is isolated from primary ovrian tumors. Xu et al. found the upregulation of Nanog was directly related to increasing Sox-2 and attenuated E-cadherin, caveolin-1, FOXO1, FOXO3a, FOXJ1, and FOXB1 mRNA expression in SKOV-3, ovarian cancer cells (Xu et al., 2012). These factors could have a role in metastasis in ovarian cancer. Together, these data suggest a role for OCSC in the transformation of the immune system from attacking tumor cells to promoting tumor progression (Figure 1).

## Targeted therapy for OCSC

**FIGURE 2:**
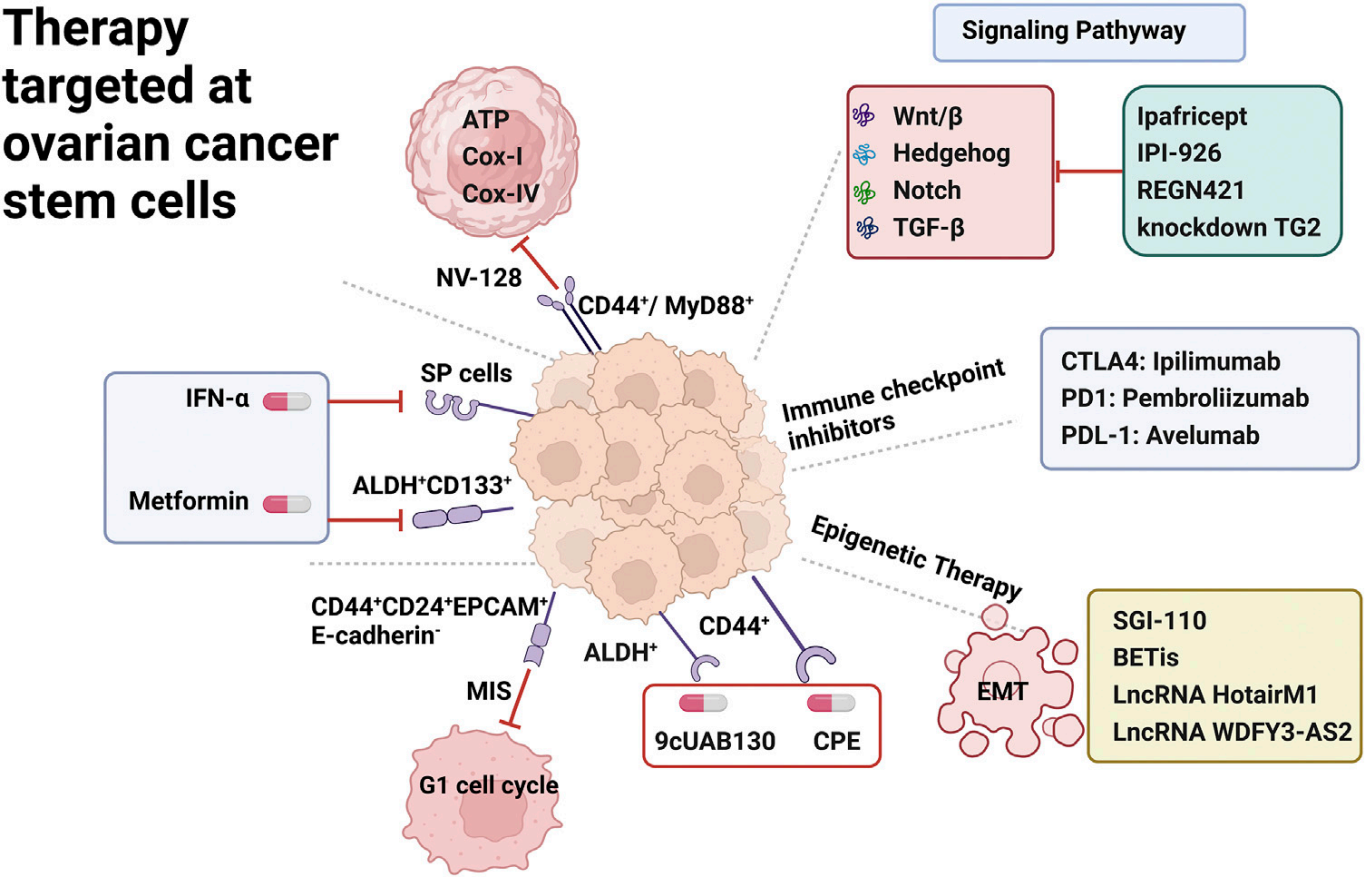
Therapy targeted at ovarian cancer stem cells. OCSC surface markers including SP Cells, ALDH^+^ CD133^+^, CD44^+^CD24^+^EPCAM^+^E-cadherin^-^, CD44^+^/MyD88^+^, CD44^+^; underlying signaling pathway including Wnt/β, Notch, Hedgehog, TGF-β; immune checkpoint inhibitor including CTLA4,PDL1,PDL-1 inhibitors; epigenetic therapy including EMT epithelial to mesenchymal transition.

One of the main challenges in the successful treatment of OC is the development of recurrence and drug-resistant disease. Even with a satisfactory response to conventional chemotherapy, nearly 70% of ovarian cancer patients relapse within 5 years and are characterized by recurrence and chemoresistance (Morand et al., 2021). Although in-depth studies of OC samples have provided insight into the alterations leading to disease pathology, it has not captured the important molecular drivers of chemoresistance and recurrent disease (Saygin et al., 2019). Targeted OCSC therapy, which is less toxic than conventional chemotherapy, is aimed at specific molecular pathways and their complex interactions to achieve therapeutic goals (Mihanfar et al., 2019). However, several therapeutic approaches for OCSCs themselves are not well established, and clinical trials results have not yet drawn constructive conclusions. Potential targets for OCSC therapy are depicted in Figure 2.

### Targeting OCSC surface markers

Considerable efforts are underway to identify therapeutic approaches that specifically target surface markers of OCSC. IFN-α exerts significant anti-proliferative and pro-apoptotic effects on primary cultures containing large numbers of SP cells. *In vitro*, IFN-α treatment resulted in a significant reduction in SP size in tumor cell lines of different origins. Furthermore, tumors established in immunocompromised mice responded more favorably to human IFN-α treatment (Moserle et al., 2008). Metformin, a type 2 diabetes drug, has been shown to selectively kill chemotherapy-resistant CSC in breast cancer cell lines (Foster et al., 2013). Similarly, metformin can limit the growth and proliferation of ovarian cancer stem cells *in vitro* and *in vivo*, reduce the percentage of ALDH^+^ CSCs, and inhibit the spheroid-forming ability of ALDH^+^ cells isolated from established cell lines and short-term patient tumor cell cultures (Shank et al., 2012). Metformin limited the growth of ALDH^+^ CSC xenografts (Shank et al., 2012). In addition, a phase II clinical trial showed that metformin also targeted ALDH^+^ CD133^+^ CSC resulting in a significant more than 2-fold reduction in CSC numbers and enhanced organismal sensitivity to cisplatin (Brown et al., 2020).

CD44^+^CD24^+^EPCAM^+^E-cadherin^-^ cells are resistant to the chemotherapeutic drug adriamycin, and stimulation with adriamycin significantly increases the number of colonies in the cells. However, these cells are sensitive to mullerian inhibiting substance (MIS), which reduces colony formation and proliferation rate of human ovarian cancer stem cells by inducing G1 cell cycle arrest and increasing cell cycle inhibitors (Meirelles et al., 2012). Novo isoflavone derivative NV-128 was found by Alvero et al. to target CD44^+^/MyD88^+^ ovarian cancer stem cells by inhibiting ATP, Cox-I and Cox-IV levels resulting in a decrease in mitochondrial function and a corresponding increase in reactive oxygen species, ultimately leading to loss of mitochondrial membrane potential and cell death. This ability to specifically target the mitochondria of chemoresistant ovarian cancer stem cell populations opens up new avenues for the treatment of ovarian cancer patients (Alvero et al., 2011). To target ALDH^+^ cells, Whitworth et al. found that novel retinoids in combination with carboplatin chemotherapy could inhibit their activity. Co-treatment of ovarian cancer cell line A2780 with the novel retinoic acid 9cUAB130 and carboplatin reversed the increase in ALDH^+^ cells observed after treatment with carboplatin alone. This finding further supports the concept that CSC survive and may proliferate in response to standard chemotherapy. In addition, injection of A2780 cells treated with a combination of retinoid and carboplatin into the lateral abdomen of thymus-free nude mice prevented their ability to become tumorigenic in immunocompromised mice (Whitworth et al., 2012). There are other studies that have similarly analyzed the ability of specific compounds to modulate OCSC chemoresistance. Resistant CD44^+^ cells derived from patient ascites or tumor samples in culture were highly sensitive to *Clostridium perfringens* enterotoxin (CPE). Importantly, in mice transplanted with chemoresistant CD44^+^ ovarian cancer stem cell xenografts, multiple intraperitoneal treatments with sublethal doses of CPE significantly inhibited ovarian cancer progression (Casagrande et al., 2011).

### Targeting metabolic processes associated with OCSC

In general, the phenotypic characterization of stem cells depends on the application of flow cytometry in combination with the measurement of functional stem cells. However, the utilization of surface markers alone does not seem to be a therapeutic tool due to many limitations such as technical challenges, tumor heterogeneity and lack of high specificity (Nagare et al., 2017). In this case, the validation of metabolic markers can integrate information from true CSC markers, allowing for more precise targeting of cancer stem cell therapy. Several researches have described that OXPHOS and mitochondria may play a vital role in CSC metabolism, as well as secondary pathways such as pentose phosphate pathway (PPP), fatty acid oxidation (Peiris-Pagès et al., 2016).

It has been shown that mutations in mitochondrial DNA (mtDNA) are associated with the development of ovarian cancer and that maintaining intact mitochondrial function is essential (Kim et al., 2015). Elevated OXPHOS activity in hyper-proliferative cells has been proven to be a leading driver of mitochondrial genomic aberrations in tumorigenesis. A research showed that CD44^+^ CD117^+^ OCSCs separated from the ascites of OC patients demonstrated accelerated glucose uptake and possessed an OXPHOS-dominated metabolic profile with higher ROS production and higher membrane electrical potential. Furthermore, these OCSCs were more sensitive to OXPHOS inhibitors *versus* CD44^+^ CD117^+^ non-OCSCs (Pastò et al., 2014). Ovarian CSC-like spheroid cells were observed to be dependent on anaerobic glycolysis and PPP. Furthermore, the quantity of glucose exploited in the PPP is substantially higher than that CSC-like spheroid cells oxidize *via* the TCA cycle (Liu et al., 2014). In addition, the oxidative branch of the pentose cycle is an efficient means of generating cytoplasmic NADPH, and high NADPH production is necessary to increase fatty acid synthesis (a NADPH-dependent process) (Liao et al., 2014). Consistent with this, previous study which used *in vitro* cell culture showed that OCSC isolated from suspended ascites changed its metabolism from glycolysis to increased fatty acid metabolism (Sato et al., 2018). In this context, pharmacological manipulation of glycolysis to prevent OCSC proliferation has proven to be an effective strategy. Metformin hydrochloride is an antidiabetic drug for treating type 2 diabetes that enhances the effect of chemotherapy by targeting CSC. It has been studied that metformin has synergistic effects with conventional chemotherapeutic drugs to minimize tumor regression rates (Brown et al., 2020). Consequently, an in-depth understanding of the metabolic processes that characterize OCSC appears to be one of the key elements to assess their potential for targeted therapy.

### Targeting underlying signaling mechanisms for OCSC

There is increasing evidence that various signaling pathways such as Wnt/β, Hedgehog, Notch, and TGF-β play an important role in the initiation of proliferation and metastasis of OCSCs. Therefore, various clinical studies on signaling pathway blockers are underway to validate their clinical significance in eliminating OCSCs and preventing recurrence. Notch signaling is essential for normal stem cell function and dysregulation of this pathway has been demonstrated in many cancers, with the identification of three genes encoding regulators of the Notch signaling pathway in ascites samples from ovarian cancer patients (South et al., 2012). Treatment of the SP fraction derived from ovarian cancer cell lines with the Notch pathway inhibitor γ-secretase resulted in a dose-dependent decrease in cell survival. Importantly, the defined SP gene expression profile was enriched in recurrent ovarian tumors. These data suggest that the Notch signaling pathway in ovarian cancer regulates OCSC survival and self-renewal as well as tumor maintenance (Vathipadiekal et al., 2012). Furthermore, alterations in Notch signaling pathway components were one of the few common mutations in a recent sequencing analysis of 489 high-grade plasmacytic human ovarian tumors (Integrated genomic analyses of ovarian, 2011). A phase I dose-escalation trial of Ipafricept (NCT01608867) found that Ipafricept (OMP-54F28), a recombinant fusion protein targeting the Wnt/β signaling pathway, was better tolerated in combination with conventional chemotherapy in patients with advanced ovarian cancer (Jimeno et al., 2017). Notch and Wnt-β signaling pathways are thought to interact to promote tumor growth through the survival of CSCs. In a phase I human study (NCT00871559) in patients with advanced ovarian cancer, Enoticumab (REGN421), a Delta-like ligand 4 (Dll4) monoclonal antibody that disrupts Notch-mediated signaling was shown to be a safe agent (Chiorean et al., 2015).

Aberrant activation of the Hedgehog signaling pathway has been implicated in the pathogenesis of multiple tumors. The Hedgehog pathway inhibitor cyclopamine and the clinically applicable derivative IPI-926 inhibit plasmacytic tumor growth. Tumor recurrence after cessation of paclitaxel and carboplatin treatment was blocked when Hedgehog inhibitors were given as consolidation therapy, suggesting that CSC-specific Hedgehog signaling may be critical for the development of recurrent disease (McCann et al., 2011).

Transforming growth factor (TGF)-β mediates epithelial mesenchymal transition (EMT) in ovarian cancer by regulating tissue transglutaminase (TG2) involved in cell proliferation, differentiation and apoptosis, and is associated with the development of CSCs. CD44^+^/CD117^+^ cells isolated from human ovarian tumors express high levels of TG2, and *in vitro* treatment of IGROV1 cells with TGF-β enhanced spheroid formation in culture and increased the number of CD44^+^/CD117^+^ cells. Targeted knockdown of TG2 in human primary ovarian tumor cells blocked their ability to form spheroids. These data suggest that targeting the TGF-β pathway may be effective in disrupting ovarian cancer progression (Cao et al., 2012).

Steg et al. examined the expression of the CSC markers ALDH1A1, CD44 and CD133 in 45 matched pairs of primary/recurrent high-grade ovarian adenocarcinomas and demonstrated that the expression of all three CSC markers was increased after completion of primary chemotherapy. Similarly the expression of Hedgehog, Notch, TGF-β and Wnt cell signaling pathway members was also enhanced after initial chemotherapy. In tumors collected from recurrent platinum-resistant patients, only CD133 was significantly increased. Knockdown of TGF-β and Hedgehog pathway components revealed a decrease in ovarian cancer cell viability. This study demonstrates that the enrichment of ovarian CSC and stem cell pathway members after initial chemotherapy suggests that ovarian cancer chemoresistance and recurrent disease are driven in part by CSC (Steg et al., 2012).

### Immune checkpoint inhibitors for OCSC

Immune checkpoint inhibitors (CPIs) are those involving the PD1/PD-L1 and CTLA-4/CD80/CD86 pathways. Various phase I/II clinical trials have been conducted on immune checkpoint inhibitors, such as anti-PDL1 Avelumab (NCT01772004) and Atezolizumab (NCT01375842), anti-PD-1 Pembrolizumab (NCT02674061) for recurrent cases of advanced ovarian cancer. However, the initial results obtained were far from satisfactory. To improve the results, various phase III clinical trials (NCT03598270, NCT02891824, NCT02659384) are underway (Borella et al., 2020). PARP1 is mainly associated with DNA damage repair, inducing DNA damage followed by the addition of poly (ADP-ribose) chains to the target molecule, PARPis with BRCA1 or BRCA2 gene mutations are particularly effective in cancer cells (Li et al., 2019). The association between PARPis and immune CPIs is a potentially successful approach for EOC treatment and several clinical trials testing this association are currently underway, such as KEYLYNK-001, FIRST, and ATHENA, but no preliminary data are yet available (McCann et al., 2011). Another possibility to improve the effectiveness of immune CPIs is to use them in combination with angiogenesis inhibitors, which can “normalize” the tumor vascular architecture and thus change the infiltration of T cells into the tumor by inhibiting VEGF and increasing the effectiveness of chemotherapy (Shrimali et al., 2010). An ongoing phase III study is enrolling newly diagnosed high-risk OC patients to evaluate pembrolizumab and bevacizumab (GOG3015) (Borella et al., 2020). In addition, phase II/III clinical trials of various tyrosine kinase inhibitors, such as Pazopanib, Nintedanib (BIBF 1120), Cediranib, and Sunitinib have shown promising results against the combination of VEGF receptor, PDGF receptor, C-tyrosine kinase, and FMS-like tyrosine kinase-3 (Paoletti et al., 2020). However, OC remains one of the few malignancies in which CPIs have not been incorporated into the approved standard of immunotherapy. This could be explained by the low tumour mutational burden. It is crucial to figure out whether OCSCs can be targeted in order to improve the efficacy of CPIs in OC.

### Epigenetic therapy for annihilation of OCSCs

Studies have shown that epigenetics may provide new strategies for the development of targeted cancer stem cell-like cells due to the important role of epigenetic regulators in the control of normal stem cell differentiation (Plumb et al., 2000). Epigenetic dysregulation promotes increased survival and plasticity in ovarian CSCs, leading to the development of their metastatic features. The DNA methyltransferase inhibitor SGI-110 effectively aided the differentiation of ALDH^+^ OCSCs, thereby restoring platinum resensitivity in ovarian cancer cell lines (Wang et al., 2014). Targeting histone deacetylase (HDAC) isoforms allows chromatin remodeling through histone acetylation, and HDAC inhibitors also block key signaling pathways associated with CSC maintenance. In addition, different HDAC isoforms can regulate the protein stability and/or activity of EMT inducible transcription factors, including HIF-1α, Stat3, Notch1, β-catenin, NF-κB, and c-Jun (Lin et al., 2018). Bromodomain and extraterminal domain (BET) is an epigenetic reader that regulates the expression of several genes involved in oncogenesis. BET inhibitors (BETis) can suppress tumorigenesis by inhibiting ALDH activity (Sarnik et al., 2021). LncRNA HotairM1 can recruit EZH2 and SUZ12 to the promoter of its target gene HOXA1, leading to histone H3K27 trimethylation and epigenetic silencing of HOXA1, promoting CSC self-renewal and tumor proliferation (Li et al., 2020). LncRNA WDFY3-AS2 promotes cisplatin-resistant and CD44+CD166+cells in ovarian cancer by regulating the hsa-miR-139-5p/SDC4 axis (Wu et al., 2021). These studies provide evidence of a cross-talk between epigenetic regulation through LncRNAs and ovarian cancer progression, suggesting a role for LncRNAs as a potential therapeutic target to prevent ovarian cancer recurrence by eliminating CSCs along with conventional therapies.

## What are the challenges for OCSC-targeted therapy

Although OCSC-targeted therapy offer a new treatment strategy for ovarian cancer patients with promising applications, it remains true that there are many problems that need to be solved and no such therapy has entered into the clinical standard of care. Below we summarize a few of the challenges that need to be overcome. (i) Lack of reliable predictive markers to assess efficacy. One of the primary difficulties in obtaining highly specific surface markers for OSCS arises from the heterogeneity between tumors. (ii) Research on ovarian CSC-focused studies has not captured the equally important molecular drivers of chemoresistance and recurrent disease. Due to the lack of samples from resistant and recurrent disease, most studies assessing their prevalence or function have been dependent on tissues that did not receive chemotherapy. (iii) The effect of immune checkpoint therapy is slow. It takes several months to show efficacy and can cause autoimmune diseases such as dermatitis, enteritis and hepatitis. (iv) Targeted inhibitors are not widely used in the treatment of ovarian cancer. Most of the inhibitors that have proven efficacy are still in the experimental stage. (v) The choice of targeted inhibitors in combination with chemotherapeutic agents is still inconclusive. The optimal timing and dose of targeted inhibitors are also controversial.

## Conclusion

There is sufficient evidence to support the presence of CSCs in ovarian tumors that can initiate tumors, participate in tumor immune escape, are resistant to chemotherapy, and produce more differentiated non-tumorigenic cells. Unfortunately, immunotherapy is not currently been approved for ovarian cancer treatment. Further development of effective therapies against CSCs requires a more detailed understanding of the various cell biological processes, the patient’s tumor grade, the interaction between cancer cells and CSCs, and the cancer-microenvironment components. Overall, recent developments in the identification of candidate therapies targeting OCSCs reflect significant advances in this area and may lead to the development of new clinical treatment strategies regarding ovarian cancer therapy.

## Abbreviations

CSC, cancer stem cells; OC, Ovarian cancer; OCSC, Ovarian cancer stem cells; TAA, tumor-associated antigen; DC, dendritic cell; MHC, major histocompatibility complex; TAM, tumor-associated macrophages; NK, Natural kille; MDSC, myeloid-derived suppressor cell; SP cell, side-population cell; ARG-1, arginase 1; NOS, inducible nitric oxide synthase; Siglec-10, sialic-acid-binding Ig-like lectin 10; CXCR4, C-X-C motif chemokine receptor four; CPIs, Immune checkpoint inhibitors.
